# Tissue-engineered blood-brain barrier models via directed differentiation of human induced pluripotent stem cells

**DOI:** 10.1038/s41598-019-50193-1

**Published:** 2019-09-27

**Authors:** Gabrielle N. Grifno, Alanna M. Farrell, Raleigh M. Linville, Diego Arevalo, Joo Ho Kim, Luo Gu, Peter C. Searson

**Affiliations:** 1 Institute for Nanobiotechnology, Johns Hopkins University, Baltimore, MD USA; 2 Department of Biomedical Engineering, Johns Hopkins University, Baltimore, MD USA; 3 Department of Materials Science and Engineering, Johns Hopkins University, Baltimore, MD USA

**Keywords:** Tissue engineering, Blood-brain barrier

## Abstract

Three-dimensional (3D) tissue-engineered models of the blood-brain barrier (BBB) recapitulate *in vivo* shear stress, cylindrical geometry, and cell-ECM interactions. Here we address four issues associated with BBB models: cell source, barrier function, cryopreservation, and matrix stiffness. We reproduce a directed differentiation of brain microvascular endothelial cells (dhBMECs) from two fluorescently labeled human induced pluripotent stem cell lines (hiPSCs) and demonstrate physiological permeability of Lucifer yellow over six days. Microvessels formed from cryopreserved dhBMECs show expression of BBB markers and maintain physiological barrier function comparable to non-cryopreserved cells. Microvessels displaying physiological barrier function are formed in collagen I hydrogels with stiffness matching that of human brain. The dilation response of microvessels was linear with increasing transmural pressure and was dependent on matrix stiffness. Together these results advance capabilities for tissue-engineered BBB models.

## Introduction

Microvessels of the blood-brain barrier (BBB) separate the bloodstream from the central nervous system (CNS)^1^. Brain microvascular endothelial cells (BMECs) form tight junctions, which restrict paracellular transport, and an array of efflux pumps and transporters, which regulate transcellular transport into the brain. The functional BBB prevents entry of the majority of pharmaceutical agents into the brain, particularly those of high molecular weight, hindering treatment strategies for CNS disease^2^, while a dysfunctional BBB is implicated in neurological disease^3,4^. BMECs generated via differentiation of human induced pluripotent stem cells (hiPSCs) are a promising cell source for *in vitro* studies of the BBB^5–8^. However, most models of the BBB are constructed in two-dimensions (2D) and are therefore unable to recapitulate shear stress, cell-ECM interactions, and cylindrical geometry characteristic of the *in vivo* brain microvasculature^9^. Three-dimensional (3D) models of the BBB that incorporate these complexities and achieve physiological barrier function have recently been developed^10–12^.

Tissue-engineered models are indispensable for studies of the BBB as they integrate spatial and temporal information on cell behavior and barrier function, similar to two-photon microscopy approaches *in vivo*. However, multiple challenges limit the application of such models: (1) The spontaneous differentiation of iPSCs remains a time-consuming step in microvessel fabrication. The differentiation generates a sub-population of BMECs which is purified by subculture on collagen IV and fibronectin-coated surfaces^6,7^. This purification step reduces cell adhesion and has restricted formation of microvessels to stiff collagen I hydrogels^11,13^. However, the use of cross-linkers to increase stiffness can limit the ability to co-culture cells in the surrounding matrix. As an alternative, the directed differentiation of dhBMECs^5^ does not involve a purification step and hence could increase the efficiency of microvessel fabrication and expand the range of matrix materials. Furthermore, extending the repertoire of cell lines to include fluorescently labeled hiPSCs is critical to enabling live-cell imaging in 3D tissue-engineered models. (2) Mimicking physiological barrier function in tissue engineered models is critical for studying clinically-relevant processes such as BBB-opening and drug delivery^9,14,15^. While recent advances have led to *in vitro* models with physiological barrier function; the ability to maintain stable barrier function within tissue-engineered models has not widely been reported^10^. This limits the utility of models for studying processes which occur over days to weeks. (3) dhBMECs do not maintain phenotype following passaging, therefore, freshly differentiated dhBMECs are required for most experiments. Recent studies have reported that cryopreservation of dhBMECs obtained by spontaneous differentiation^16^ show similar barrier properties as freshly differentiated cells. Cryopreservation has not been validated for 3D BBB models, but would increase fabrication efficiency. (4) As the human brain is highly cellular (70–85% by volume), selection of an appropriate extracellular matrix (ECM) for 3D models is challenging^17^. As a proxy for the cellular components, 3D models of the BBB commonly utilize ECM proteins not present in the brain, including collagen I^11,18–22^ and fibrin^10,23^. In previous work we demonstrated physiological barrier function in a tissue-engineered microvessel model within a stiff collagen matrix^11,13^. However, the role of matrix stiffness on the structure and function of 3D blood-brain barrier microvessels has not been further explored.

In this paper we generate tissue-engineered 3D models of the human BBB which address these key challenges: (1) cell source – we reproduce a recently reported directed differentiation of dhBMECs^5^ using fluorescently-tagged hiPSCs which enable live-cell imaging, (2) barrier function – we demonstrate stable and physiological permeability of solutes over six days, (3) cryopreservation – fresh and cryopreserved dhBMECs display similar barrier function in 2D and 3D models, and (4) the role of matrix stiffness in BBB structure and function – the solute permeability of microvessels remains low across matrix stiffness ranging from 0.3–3.3 kPa, while the structural stability and dilation response were stiffness-dependent. Together, these advances in cell culture and live-cell imaging establish tissue-engineered microvessels as a versatile platform for studies of BBB function/dysfunction and highlight the importance of matrix stiffness in BBB microvessel behavior.

## Results and Discussion

### Differentiation and characterization of hiPSC-derived BMECs

**Figure 1:**
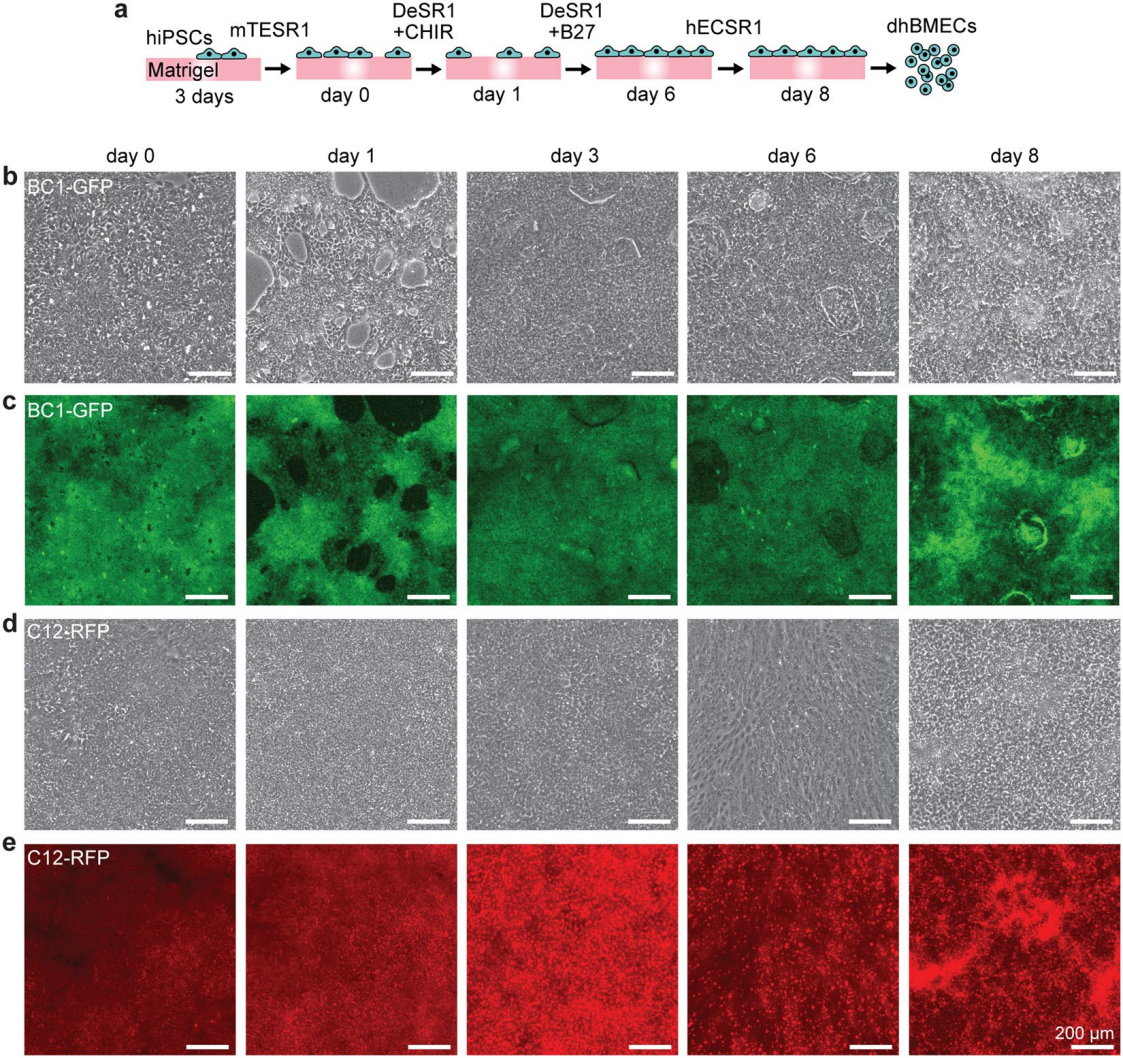
Directed differentiation of human brain microvascular endothelial cells (dhBMECs) from two induced pluripotent stem cell lines (BC1-GFP and C12-RFP). (**a**) Schematic illustration of differentiation over eight days. (**b**,**c**) Phase and fluorescence images of a representative BC1-GFP differentiation. (**d**,**e**) Phase and fluorescence images of a representative C12-RFP differentiation. Cytoplasmic fluorescence is maintained across both differentiations.

To produce a stable cell source for the formation of tissue-engineered BBB microvessels, we adapted a previously reported protocol^5^ for the directed differentiation of two fluorescently-labeled human induced pluripotent stem cell lines (hiPSCs), BC1-green fluorescent protein (BC1-GFP) and C12-red fluorescent protein (C12-RFP), into brain microvascular endothelial cells (dhBMECs). The directed differentiation uses a multistep protocol over the course of eight days (Fig. 1a) to drive differentiation of hiPSCs first towards an intermediate mesoderm stage, then towards brain endothelial cell specificity via canonical Wnt pathway agonism and the addition of neuronal and retinoic acid signaling supplements^5^. Daily phase contrast and epifluorescence imaging of cell cultures was used to monitor the progress of the differentiation.

We found that the concentration of CHIR99021 and starting confluency had synergistic effects on BC1-GFP differentiation outcomes (Supplementary Fig. S1). CHIR99021 has previously been found to be cytotoxic to hiPSCs with reduced S/G2/M cell-cycle phases, underlying issues with cardiac differentiation inconsistencies and low reproducibility^24^. 6 μM CHIR99021 treatment resulted in the highest mean transendothelial electrical resistance (TEER), matching the concentration previously used for directed differentiation of BMECs (Supplementary Fig. S1a)^5^. At this concentration, hiPSC seeding density was found to be critical to the success of the differentiation, and we determined that hiPSCs seeded at a density between 20,000–30,000 cells cm^−2^ resulted in the highest TEER (Supplementary Fig. S1b).

To further understand the variability of the directed differentiation across cell lines, we tracked cell confluence for both BC1-GFP and C12-RFP differentiations. Both cell lines reached ~95% confluence in three days when seeded at 20,000 cells cm^−2^. The addition of increasing concentrations of CHIR99021 resulted in a dose-dependent decrease in cell confluence for BC1-GFPs (r^2^ = 0.56) (Supplementary Fig. S2a,b). In contrast, the confluence of C12-RFPs was independent of concentration, suggesting that CHIR00921 can display cell line-dependent effects (Supplementary Fig. S2a,b). Additionally, changes in confluency were correlated with success of the differentiation (as measured by TEER) only for BC1-GFPs (r^2^ = 0.38) (Supplementary Fig. S2c). Under these optimized conditions, BC1-GFP and C12-RFP lines maintained cytoplasmic fluorescence throughout the differentiation (Fig. 1b–e). While at the early stages of the differentiation the fluorescence was relatively uniform, during the latter stages the fluorescence intensity was more variable within each culture well due to differences in cell density (Fig. 1b–e).

**Figure 2:**
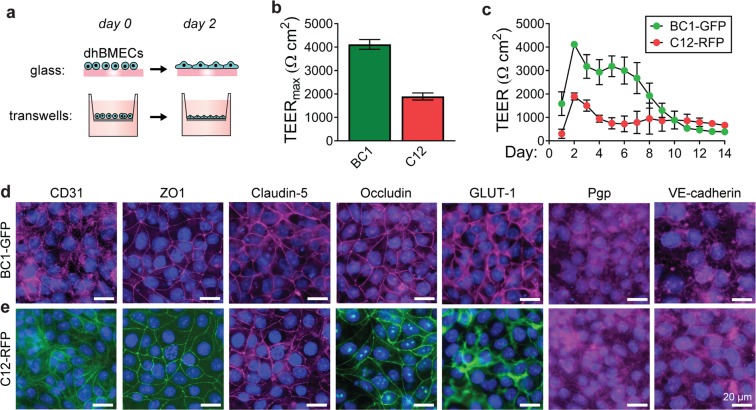
Transendothelial electrical resistance (TEER) and immunocytochemistry of dhBMECs derived from the BC1-GFP and C12-RFP iPSC lines. (**a**) Schematic illustration of timeline for cell seeding following differentiation. (**b**) Maximum TEER for each cell line. (**c**) TEER values for each cell line over two weeks. TEER data represent mean ± SEM for 3–4 technical replicates across 3 independent differentiations for BC1-GFPs and 4 independent differentiations for C12-RFPs. (**d**,**e**) Fluorescence images of representative dhBMEC monolayers stained for CD31, ZO-1, claudin-5, occludin, GLUT-1, p-glycoprotein (Pgp), and VE-cadherin (violet or green). Nuclei (blue).

On day eight of the differentiation, dhBMECs were singularized and seeded onto Matrigel-coated Transwells to monitor TEER (Fig. 2a). TEER peaked two days after seeding at 4,118 ± 119 Ω cm^2^ and 1,897 ± 76 Ω cm^2^ for BC1-GFP and C12-RFP lines, respectively (Fig. 2b). The difference in TEER between BC1-GFP and C12-RFP is similar to differences reported across three stem cell lines previously used to demonstrate this differentiation approach^5^. The cell seeding density was the same for both cell lines, and there was no difference in cell density in confluent monolayers as inferred from immunocytochemistry images of nucleus density (p = 0.698). BC1-GFP dhBMECs maintained TEER values above 2,000 Ω cm^2^ for seven days, and C12-RFP dhBMECs maintained TEER values above 1,500 Ω cm^2^ for two days (Fig. 2c). Both lines maintained values above 500 Ω cm^2^ for over ten days (Fig. 2c). TEER is a commonly used method to assess barrier integrity, with physiological values in the range from 1,500–8,000 Ω cm^2^ ^25–28^. Previous studies have shown that dhBMECs obtained using the standard 2-step spontaneous differentiation show an increase in sodium fluorescein permeability for TEER values ≤ 500 Ω cm^2^, implying that barrier function is maintained above this threshold^29^. These results demonstrate that BC1-GFP and C12-RFP dhBMECs can maintain long-term barrier function without daily media changes or the addition of other supporting cell types (i.e. astrocytes or pericytes). Retention of physiological barrier function is critical for studies requiring long incubation periods, such as studies of neurodegenerative protein aggregate accumulation.

To determine whether cells properly express characteristic BBB endothelial markers, we stained confluent monolayers for tight junction markers, nutrient transporters, and efflux transporters (Fig. 2a). Directed differentiation of BMECs from both the BC1-GFP and C12-RFP sources produced cells with localized claudin-5, occludin, and zona occludens-1 (Fig. 2d). Additionally, we confirmed the junctional expression of endothelial cell markers CD31 and VE-cadherin, and global expression of glucose transporter 1 (GLUT-1) and the p-glycoprotein (P-gp) efflux pump (Fig. 2d). These markers have previously been reported in other dhBMEC differentiations^5–7^. The relative contributions of endothelial markers and tight junction proteins on barrier function are not well understood; however, claudin-5 is thought to be critical to achieve physiologically high TEER values^3,30,31^.

### hiPSC-derived BMECs form functional microvessels

**Figure 3:**
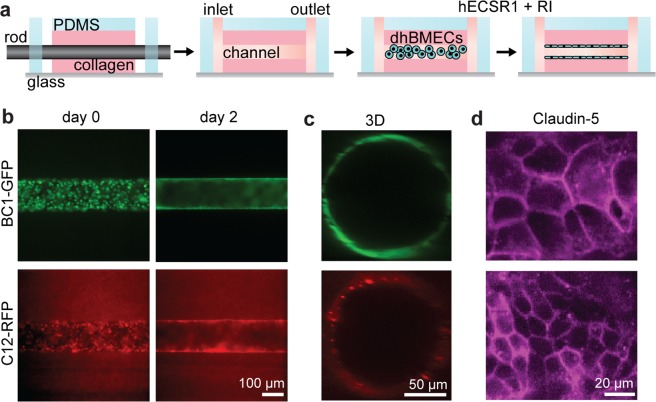
Formation and live-cell imaging of 3D blood-brain barrier microvessels. (**a**) Schematic illustration of microvessel fabrication. (**b**) Formation of a confluent monolayer occurs within two days post-seeding. (**c**) Confocal reconstructions demonstrate lumen formation on day 2. (**d**) Immunocytochemistry of the tight junction protein claudin-5 at the bottom microvessel plane on day 2. Microvessels were formed in 7 mg mL^−1^ collagen I matrix cross-linked with genipin and perfused at 1 dyne cm^−2^.

In previous work, we showed that tissue-engineered microvessels formed using dhBMECs differentiated using a two-step protocol exhibited physiological barrier function in cross-linked 7 mg mL^−1^ collagen gels^11,13^. This approach relies on the isolation of brain endothelial cells from a heterogeneous cell population through selective adhesion, achieved by sub-culturing terminally differentiated cells onto collagen IV and fibronectin-coated dishes prior to seeding into microvessels. To assess the barrier function of dhBMECs from the directed differentiation, we formed functional microvessels using a templating method reported previously (Fig. 3a)^11,13,32^. dhBMECs from BC1-GFPs and C12-RFPs were detached from culture dishes and immediately seeded within 150 μm diameter channels in collagen hydrogels.

First, we assessed microvessels formed in 7 mg mL^−1^ collagen I gels cross-linked with genipin. Following seeding, dhBMECs formed a confluent monolayer over two days without the need for a sub-culture selection step (Fig. 3b,c). Monolayer formation was consistent with BBB microvessels formed using the 2-step differentiation. Microvessels were subjected to a shear stress of approximately 1 dyne cm^−2^, within the range of typical values for *in vivo* post-capillary venules (PCVs) of 1–4 dyne cm^−2^ ^33–37^.

**Figure 4:**
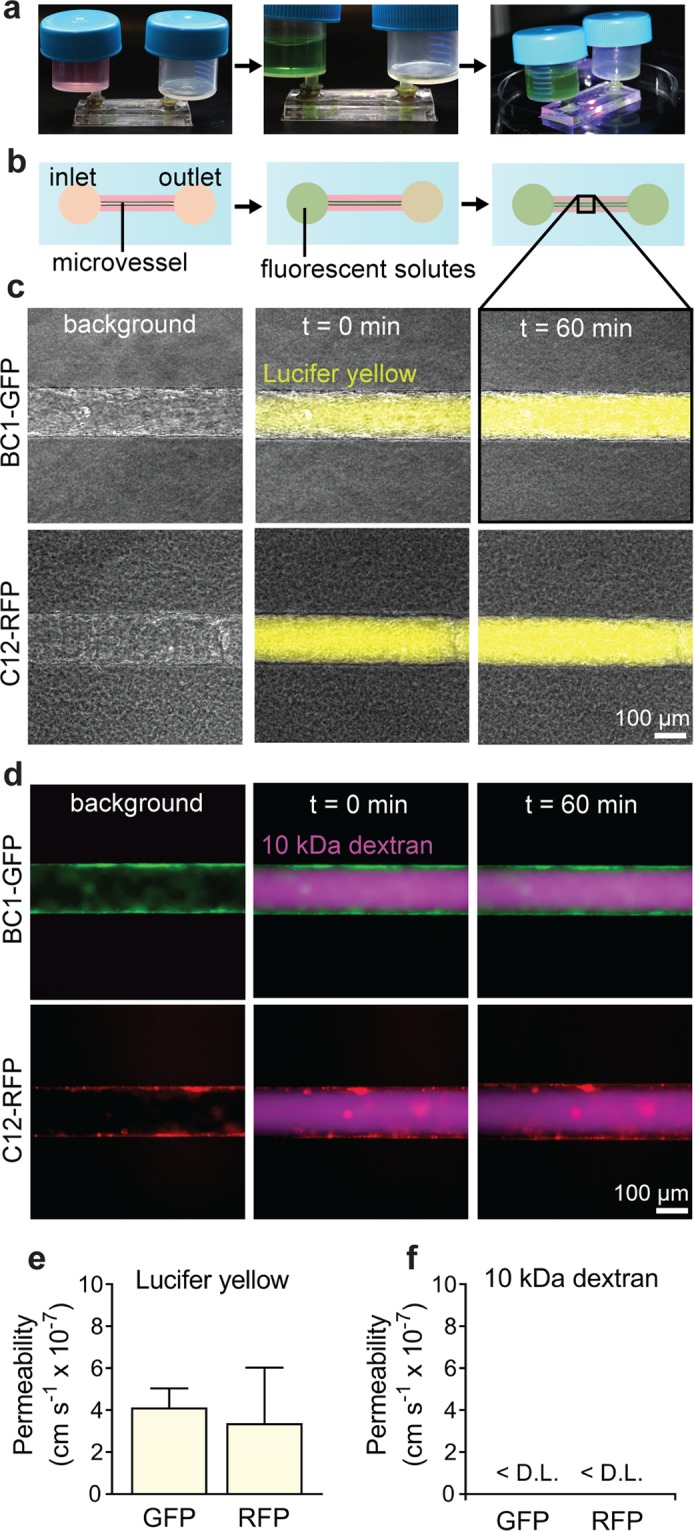
Permeability of 3D blood-brain barrier microvessels. (**a**) Microvessels were maintained under continual perfusion (~1 dyn cm^−2^) and fluorescent dyes introduced into the flow loop to quantify permeability. (**b**) Schematic illustration showing timeline for permeability experiments. Representative images are taken prior to introducing the solute, when the solute appears in the microvessel lumen, and after 60 minutes. (**c**,**d**) Representative phase / fluorescence images of Lucifer yellow and 10 kDa dextran permeability. (**e**,**f**) Quantification of Lucifer yellow and 10 kDa dextran permeability. Microvessels were formed in 7 mg mL^−1^ collagen I matrix cross-linked with genipin. Data represent mean ± SEM for 3 microvessels from 3 independent differentiations. D. L. detection limit.

BBB microvessels expressed claudin-5 localized at cell-cell junctions (Fig. 3d). Claudin-5, the most enriched tight junction protein of the BBB, is a critical regulator of barrier function, while its dysfunction is associated with loss of neurological function^3^. To further evaluate barrier function, microvessels were simultaneously perfused with two different size fluorescent probes: Lucifer yellow (LY) and 10 kDa Dextran (Fig. 4a,b). Permeability was obtained from analysis of fluorescence intensity profiles in microvessels from three independent differentiations (Fig. 4c,d). The permeability of Lucifer yellow in BC1-GFP and C12-RFP microvessels was 4.13 ± 0.91 and 3.38 ± 2.65 × 10^−7^ cm s^−1^, respectively (Fig. 4e). These values are similar to values previously reported in BBB microvessels using the 2-step spontaneous differentiation across multiple hiPSCs^11^, and are close to *in vivo* values in rat brain post-capillary venules (~1–2 × 10^−7^ cm s^−1^)^38^. Similar values were also obtained for BC1-derived dhBMECs and Madin-Darby Canine Kidney (MDCK) cells in 2D transwells, suggesting that 3D culture is not required to achieve physiological barrier function^6,39^.

The permeability of 10 kDa dextran in both BC1-GFP and C12-RFP dhBMEC microvessels was below our detection limit (Fig. 4f), similar to previous results with BBB microvessels using the 2-step differentiation. The detection limit of 10 kDa dextran permeability in genipin cross-linked hydrogels is ~1 × 10^−7^ cm s^−1^ due to photobleaching of hydrogel autofluorescence resulting from the cross-linker.^11^ Studies in zebrafish have shown that 10 kDa dextran does not enter the brain^40^; however, there remain discrepancies across *in vivo* studies concerning quantification of BBB permeability^9^.

In 2D, TEER values for BC1-GFP dhBMECs remained above 2,000 Ω cm^2^ for seven days. To confirm that 3D microvessels retained barrier function over time, we repeated permeability measurements of BC1-GFP microvessels on day six. Lucifer yellow permeability in BC1-GFP microvessels was 5.02 ± 0.94 × 10^−7^ cm s^−1^ on day six, not statistically different from day two (4.13 ± 0.91 cm s^−1^; p > 0.05). In addition, the permeability of 10 kDa dextran remained below the detection limit, indicating preservation of barrier function. These results suggest that barrier function can be maintained in 3D models without need for co-culture with supporting cell types or additional factors. Recently, we found that the incorporation of pericytes into BBB microvessels does not reduce solute permeability^41^.

### hiPSC-derived BMECs retain BBB function following cryopreservation

In tissue engineering, cell cryopreservation can eliminate the need to perform unique differentiations before each experiment. Cryopreserved dhBMECs have been shown to maintain BBB phenotype when thawed in media containing Rho-associated protein kinase inhibitor Y27632 (ROCK inhibitor)^16^. A working stock of cryopreserved dhBMECs enables scale-up, higher throughput, and can reduce batch-to-batch variability between experiments. BC1-GFP dhBMECs, which maintained higher TEER values than C12-RFP dhBMECs, were cryopreserved in liquid nitrogen (see *Materials and Methods* for details) for 11–120 days. The phenotype and barrier function of cryopreserved BC1-GFP dhBMECs was assessed and compared to non-cryopreserved controls using 2D and 3D functional assays

**Figure 5:**
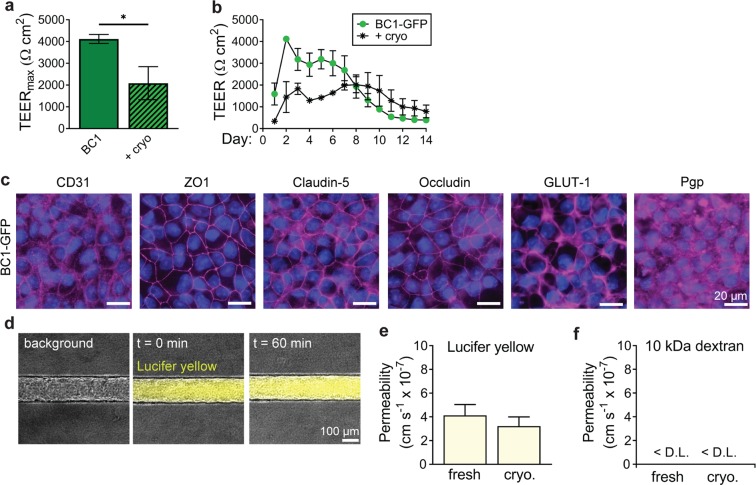
Utilization of cryopreserved dhBMECs in 2D and 3D BBB models. (**a**,**b**) Maximum TEER and time course over two weeks. Data represent mean ± SEM for 3 technical replicates over 3 independent differentiations. (**c**) Immunocytochemistry of cryopreserved BC1-GFP dhBMECs. (**d**) Representative phase / fluorescence overlays of Lucifer yellow permeability. (**e**,**f**) Permeability of Lucifer yellow and 10 kDa dextran conducted on cryopreserved BC1-GFP dhBMECs on day 2 following seeding. Data represent mean ± SEM for 3 microvessels from 3 independent differentiations. D. L. detection limit.

Cryopreserved BC1-GFP dhBMECs were 85–95% viable upon thawing (measured via Trypan blue exclusion). The morphology of thawed dhBMECs was indistinguishable from newly differentiated cells upon seeding onto glass surfaces. Cryopreserved dhBMECs achieved peak TEER values three days after seeding into Transwells (seeding conditions were identical for both cryopreserved and fresh cells besides supplementation of ROCK inhibitor), while fresh cells typically peaked two days after seeding. Confluent monolayers of cryopreserved dhBMECs exhibited TEER values above 1,000 Ω cm^2^ for more than ten days (Fig. 5b), and a peak TEER of 2,087 ± 438 Ω cm^2^ (Fig. 5a). This is within the physiological range, but lower compared to non-cryopreserved controls (peak TEER of 4,118 ± 119 Ω cm^2^; p = 0.011). Cryopreserved dhBMECs also retained expression and localization of critical tight junction, efflux, and transporter proteins comparable to non-cryopreserved controls (Fig. 5c).

We next incorporated cryopreserved dhBMECs into tissue-engineered microvessels with in 7 mg mL^−1^ collagen I gels cross-linked with genipin at the same density as microvessels formed with non-cryopreserved cells (20 × 10^6^ cells mL^−1^). Microvessels with freshly differentiated dhBMECs reached confluence one to two days after seeding, whereas the time to confluence was two to three days with cryopreserved cells, possibly due to reduced cell proliferation. However, the resulting microvessel morphology was indistinguishable to those formed with non-cryopreserved dhBMECs. Barrier function was assessed from permeability measurements following microvessel formation. The permeability of Lucifer yellow in cryopreserved dhBMEC microvessels was 3.22 ± 0.77 × 10^−7^ cm s^−1^ (Fig. 5d), not statistically different from the non-cryopreserved microvessels on day 2 (4.13 ± 0.91 × 10^−7^ cm s^−1^; p > 0.05). The permeability of 10 kDa Dextran was below the detection limit (Fig. 5f), the same as for non-cryopreserved microvessels.

### Extracellular matrix stiffness defines BBB microvessel survival, morphology, and dilation

The ECM of the brain is comprised of hyaluronic acid, lecticans, proteoglycan link proteins, and tenascins^42,43^. However, the mechanical properties of brain tissue are strongly determined by the high cell density, making hydrogels comprised only of these ECM components poor models of the native brain. For example, hydrogels fabricated from only hyaluronic acid are not mechanically strong enough to support patterning of channels (data not shown). Thus, to accurately model the brain ECM, either large volumes of cells must be incorporated or a relatively inert matrix material must be selected to provide sufficient structural support^9^. As a result, 3D models of the BBB are commonly generated using ECM proteins not found in the brain^10–12,18–23^. In previous work we used genipin cross-linked 7 mg mL^−1^ collagen I gels to form microvessels^11,13^.

Previous measurements of brain tissue have reported elastic moduli between 0.5–4.5 kPa^44–49^. The origins of this broad range are thought to be due to differences in brain region^47^, testing methods^50^, and preparation techniques^51^. Therefore, to enable direct comparison of our models and *in vivo* conditions, we obtained elastic moduli for mouse brain and hydrogels from stress - strain curves in compression^52,53^. We tested four matrix conditions: (1) 7 mg mL^−1^ collagen I cross-linked with genipin, (2) 7 mg mL^−1^ collagen I, (3) 5 mg mL^−1^ collagen I, and (4) 3 mg mL^−1^ collagen I.

**Figure 6:**
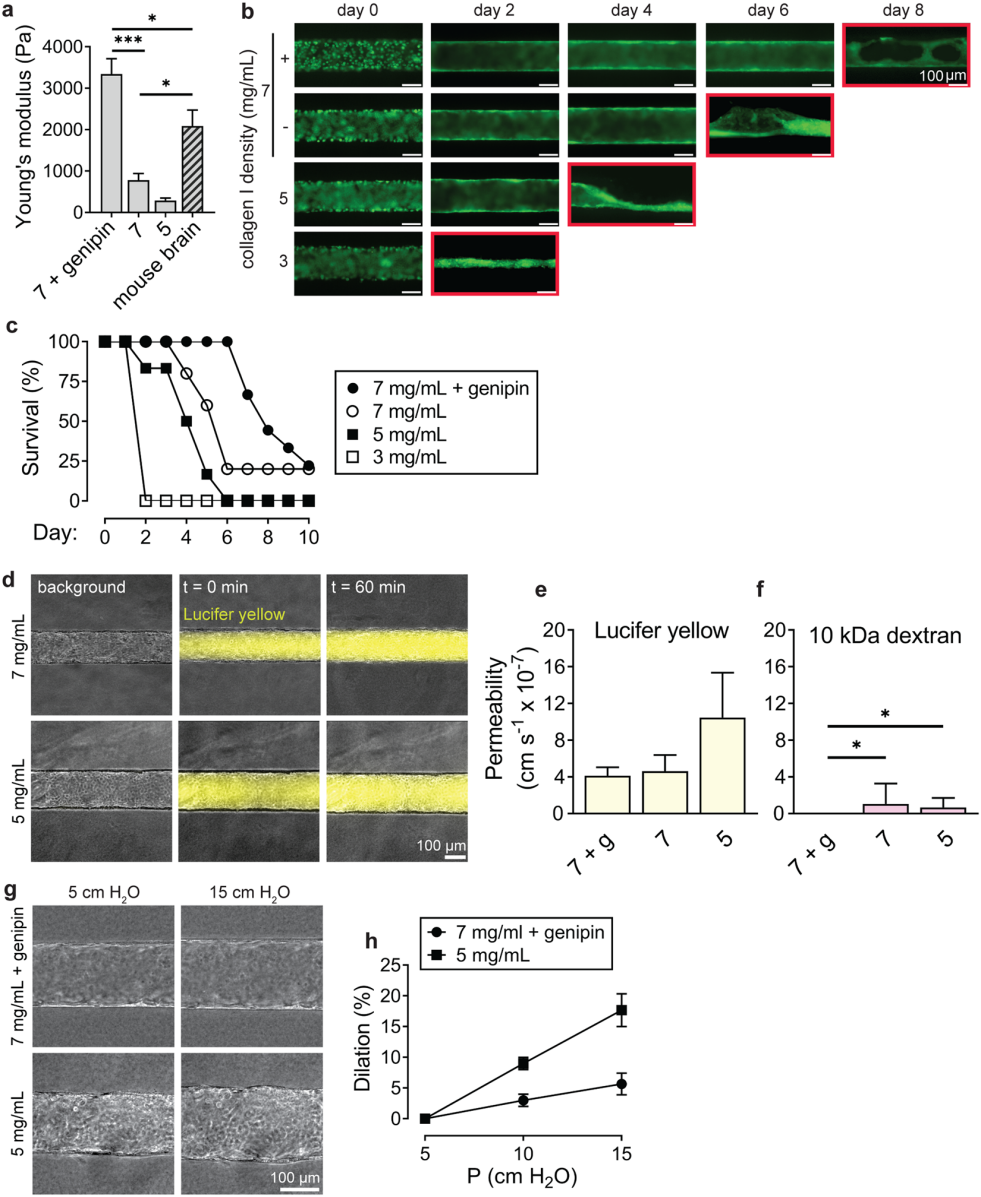
Role of ECM stiffness on structure and function of blood-brain barrier microvessels. (**a**) Comparison of Young’s modulus for bulk hydrogels: (1) 7 mg mL^−1^ collagen cross-linked with genipin, (2) 7 mg mL^−1^ collagen I, and (3) 5 mg mL^−1^ collagen I, and mouse brain. Data represent mean ± SEM for at least 8 bulk gels across 3 independent preparations, and 5 independent mouse samples. (**b**) Representative time course images of microvessels for each matrix condition. Monolayers on the softest gel condition, 3 mg mL^−1^ type I collagen, failed to reach confluence by day 2; such vessels were subsequently failed by collapse of the lumen, as indicated by the red outline on the corresponding time course image. (**c**) Survival (%) of microvessels over time based on characterization of confluent endothelium and continuous perfusion. Data represents longevity for at least five microvessels across at least 3 independent differentiations. (**d**) Representative phase/fluorescence overlays of Lucifer yellow permeability of microvessels in 7 mg mL^−1^ and 5 mg mL^−1^ collagen I on day 2 following seeding. (**e**,**f**) The influence of matrix stiffness on permeability of Lucifer yellow and 10 kDa dextran permeability on day 2. Data represent mean ± SEM for 3 microvessels from at least 2 independent differentiations. (**g**,**h**) Dilation of microvessels in response to changes in transmural pressure in 7 mg mL^−1^ microvessels cross-linked with genipin and 5 mg mL^−1^ microvessels. Under baseline conditions, the head was 5 cm water corresponding to a transmural pressure of approximately 0.05 Pa. Data represent mean ± SEM for 3 microvessels from 2 independent differentiations.

The Young’s modulus of the bulk gels increased with increasing collagen density (Fig. 6a). The moduli for the collagen I gels were 3.3 ± 0.4 kPa for 7 mg mL^−1^ cross-linked gels, 0.8 ± 0.2 kPa for 7 mg mL^−1^ gels, and 0.3 ± 0.2 kPa for 5 mg mL^−1^ gels. 3 mg mL^−1^ collagen I hydrogels were not sufficiently stiff for unconfined bulk mechanical testing. Genipin cross-linking increased the stiffness of 7 mg mL^−1^ gels by about 4-fold (p < 0.001), similar to previous reports^54^. For comparison, we measured the stiffness of resected mouse brain using the same method. The Young’s modulus for mouse brain was 2.1 ± 0.4 kPa, between values of 7 mg mL^−1^ and 7 mg mL^−1^ genipin cross-linked collagen I. Based on the elastic moduli, 7 mg mL^−1^ and 7 mg mL^−1^ genipin cross-linked collagen I are reasonable choices for emulating the stiffness of the brain.

Changes in brain stiffness, as measured using magnetic resonance elastography (MRE), are possible biomarkers for disease and aging^48,49^. Decreases in stiffness are observed during Alzheimer’s disease, but still are between values for 7 mg mL^−1^ cross-linked and non-crosslinked collagen I (2.40–2.51 kPa)^48^. The effect of changes in brain stiffness on blood-brain barrier structure and function is not known; however, changes in basement membrane are thought to alter brain function^55,56^.

To assess the adhesion and barrier function of dhBMECs over a range of ECM stiffness, microvessels were formed from non-cryopreserved BC1-GFP dhBMECs. In previous work, we used 7 mg mL^−1^ collagen I gels cross-linked with genipin to form microvessels^13^. However, the use of genipin poses multiple challenges for tissue-engineering, including the difficulty in culturing cells within highly cross-linked matrices and the cytotoxicity of unreacted cross-linker^57^. Reliable formation of BBB microvessels without the use of chemical cross-linkers would expand model complexity.

Here, dhBMECs adhered uniformly for all four matrix conditions and a confluent monolayer was formed within two days (Fig. 6b). Microvessels were formed in 3 mg mL^−1^ collagen I but did not remain perfusable due to collapse of the endothelium (Fig. 6b). Microvessels were considered viable as long as the lumen cross-section was maintained throughout the length of the microvessel, enabling continuous perfusion (Fig. 6c). BBB microvessels formed in high stiffness matrices displayed superior longevity (Fig. 6b,c). Microvessels formed in cross-linked 7 mg mL^−1^ collagen I maintained structure and fluid perfusion for eight days on average, while microvessels in 5 mg mL^−1^ collagen I were stable for four days on average. Increased transmural pressure also plays a role in microvessel stability^58^; here microvessels were maintained under 5 cm H_2_O, but increased pressures could be used to prevent collapse in softer hydrogels.

To assess the effect of hydrogel stiffness on BBB function we measured the permeability of fluorescent solutes in BC1-GFP microvessels formed in 7 mg mL^−1^ genipin cross-linked gels, 7 mg mL^−1^ gels, and 5 mg mL^−1^ collagen I gels. The values of Lucifer yellow permeability were not statistically significant from one another (p > 0.05) (Fig. 6d,e). The permeabilities of 10 kDa dextran in microvessels formed in 7 mg mL^−1^ and 5 mg mL^−1^ gels were 1.063 ± 2.208 and 0.667 ± 1.039 × 10^−7^ cm s^−1^, respectively (Fig. 6f). As described above, photobleaching of autofluorescence in genipin cross-linked gels resulted in a detection limit of about 1 × 10^−7^ cm s^−1^, however, the detection limit was lower in the non-crosslinked gels^11^.

To assess the dilation response of the stiffest (cross-linked 7 mg mL^−1^) and softest (5 mg mL^−1^) hydrogels in which stable microvessels were formed, the transmural pressure was increased by increasing the height between the fluid inlet and outlet reservoirs. We observed the response to 2-fold and 3-fold increases in transmural pressure above the baseline head of 5 cm H_2_O (approximately 0.05 Pa) (Fig. 6g). Dilation of the microvessels increased linearly with transmural pressure (r^2^ = 0.99 for both conditions). For microvessels in cross-linked 7 mg mL^−1^ gels, a 3-fold increase in transmural pressure resulted in a ~6% increase in diameter (Fig. 6h). For 5 mg mL^−1^ microvessels, a 3-fold increase in transmural pressure resulted in ~18% dilation (Fig. 6h). The response to increasing transmural pressure was significantly larger for the 5 mg mL^−1^ microvessels compared to the cross-linked 7 mg mL^−1^ microvessels (p < 0.001).

Neurovascular coupling, the process by which cerebral blood flow is matched to neuronal metabolic demand, is controlled by dilation of arterioles directly upstream from capillaries^59^. Relaxation and constriction of smooth muscle cells (SMCs) mediates these changes in the brain. Physiological arteriolar dilation ranges from about 10–30%; however, the diameter of these arterioles is typically less than 20 μm^60,61^. Autoregulation, the process by which tissue perfusion is maintained constant, is also controlled by changes in arterial diameter^62,63^. Autoregulation is maintained through multiple mechanisms including myogenic responses where vasoconstriction (mediated by SMCs) occurs in response to increases in transmural pressure (and vice versa)^64^. In arterioles of skeletal muscle in rats, a 3-fold decrease in perfusion pressure results in ~20% dilation (increase in diameter from ~85 μm to ~100 μm). However, when the SMC response is abolished (using treatment with Ca^2+^ free solution), a 3-fold decrease in perfusion pressure results in ~25% constriction (decrease in diameter from ~135 μm to ~100 μm)^65^. Additionally, in the absence of SMCs, changes in diameter were observed to be roughly linear in the same fold-change regime as studied here^65^. These studies suggest that our 5 mg mL^−1^ microvessels display dilation behaviors similar to *in vivo* arterioles devoid of SMCs. Incorporation of SMCs into tissue-engineered microvessels could recapitulate myogenic autoregulation of flow in response to transmural pressure changes.

## Conclusions

We address four key challenges associated with fabrication of tissue-engineered BBB models: cell source, barrier function, cryopreservation, and matrix stiffness. Functional microvessels formed from dhBMECs obtained from the directed differentiation of two iPSC lines showed expression of BBB markers and maintain physiological barrier function. Similarly, functional microvessels with physiological barrier function were formed from cryopreserved dhBMECs, eliminating the need for performing differentiation prior to model fabrication. Microvessels with physiological barrier function were formed in gels with stiffness ranging from 0.3–3.3 kPa, spanning the stiffness of mouse brain (~2 kPa). The dilation response of microvessels was linear with transmural pressure and was dependent on matrix stiffness, enabling simulation of the cardiac cycle.

## Materials and Methods

### hiPSC culture, differentiation and imaging

Human induced pluripotent stem cells (hiPSCs) were maintained on Matrigel-coated tissue-culture treated six-well culture plates (Corning) in mTeSR1 medium (Stem Cell Technologies). Two iPSC lines were used in this study: the BC1-GFP^66^ line was derived from a 46 year old male and the C12-RFP^67^ line derived from a newborn male. Since the research does not involve humans or animals, and the iPS lines are de-identified, no IRB approval was required. hiPSCs were maintained between passages P50-P70 and were passed using Versene (ThermoFisher). Prior to differentiation, hiPSCs were singularized using warm Accutase (Invitrogen) and plated onto Matrigel-coated six-well culture plates in mTeSR1 supplemented with 10 μM Rho-associated protein kinase inhibitor Y27632 (ROCK inhibitor; ATCC) at a density between 10,000 and 50,000 cells cm^−2^ (see Supplementary Fig. S1 for optimization of seeding density). Cell confluency was measured in ImageJ using the phase contrast microscopy segmentation toolbox (PHANTAST)^68^.

Directed differentiation of hiPSCs was adapted from a previously reported protocol^5^. Briefly, hiPSC colonies were expanded for three days in mTeSR1, then were treated with CHIR99021 (Selleckchem) at concentrations between 1–6 μM (see Supplementary Fig. S1 for optimization of concentration) in DeSR1: DMEM/F-12 (Life Technologies), 1% non-essential amino acids (Life Technologies), 0.5% GlutaMAX (Life Technologies), 0.1 mM beta-mercaptoethanol (Sigma) to initiate differentiation (day zero). After one day, the medium was changed to DeSR2: DeSR1 plus 1 × B27 (ThermoFisher) and changed daily for five days. On day six, the medium was switched to hECSR1: hESFM (Life Technologies) supplemented with 20 ng mL^−1^ bFGF (R&D Systems), 10 μM all-trans retinoic acid (Sigma) and 1 × B27. The medium was not changed for 48 hours.

hiPSC cultures were imaged daily using phase contrast microscopy and fluorescence microscopy using FITC or Texas Red filters. 10.2 mm × 7.65 mm images were acquired on an inverted microscope (Nikon Eclipse TiE) using a 4 × objective (Nikon) with epifluorescence illumination provided by an X-Cite 120LEDBoost (Excelitas Technologies).

### Cell harvesting and characterization (TEER and immunocytochemistry)

On day eight of the differentiation, cells were singularized using warm Accutase for one hour on a shaker at 100 rpm. Corning® Transwell® polyester membrane cell culture inserts (1.12 cm^2^ area, 0.4 µm pore size) were seeded at 1 × 10^6^ cells cm^−2^, while eight-chambered borosilicate cover glass wells (Lab Tek) were seeded at 0.8 × 10^6^ cells cm^−2^. All surfaces were coated overnight with 100 μg mL^−1^ growth factor-reduced Matrigel (Corning) in DMEM/F12. Cells were maintained in two-dimensional culture assays using hECSR1 for 24 hours then switched to hECSR2: hESFM supplemented with 1 × B27. Transwell cultures were maintained for 14 days, without further media changes. TEER was recorded daily as previously described^6^.

Immunocytochemistry was conducted two days after seeding dhBMECs onto borosilicate cover glass slides. Briefly, cells were first rinsed with room-temperature phosphate-buffered saline (PBS; ThermoFisher), then fixed using ice-cold methanol for 15 minutes, then blocked for 30 minutes in PBS with 10% normal goat serum (Cell Signaling Technology) and 0.3% Triton X-100 (Millipore Sigma). Primary antibodies were diluted in blocking buffer and incubated on cells overnight at 4 °C (see Supplementary Table S1 for details). After washing three times with PBS, cells were incubated with Alexa Flour-647 and/or Alexa Flour-488 secondary antibodies (Life Technologies) diluted 1:200 in blocking buffer for one hour at room temperature. To localize nuclei, cells were treated with Fluoromount-G with DAPI (Invitrogen). Images were acquired on an inverted microscope (Nikon Eclipse TiE) using a 40× objective (Nikon), and NIS Advanced Research software (Nikon), with epifluorescence illumination provided by an X-Cite 120LEDBoost (Excelitas Technologies).

### Microvessel formation

Microvessels were formed within a rectangular channel in a polydimethylsiloxane (PDMS; Sylgard 184, Dow Corning) housing with dimensions 1 cm (length) × 1.75 mm (width) × 1 mm (height) formed within an aluminum mold. The PDMS housing was attached to a glass slide following plasma treatment and treated with trimethoxysilane (Sigma) prior to gelling to reduce bubble formation between the PDMS and collagen gel. Neutralized rat tail type I collagen gel (Corning) ranging from 3 to 7 mg mL^−1^ was introduced into the PDMS housing around a 150 μm diameter super-elastic nitinol wire (Malin Co.) which served as a template for the microvessel. Collagen solutions were neutralized on ice and then gelled for 30 minutes at 37 **°**C. Prior to the removal of the template rod, the addition of 2% agarose to both sides of the collagen gel prevented delamination. To increase stiffness, 7 mg mL^−1^ gels were crosslinked using 20 mM genipin (Wako Biosciences) for two hours^54^; devices were then perfused with PBS for at least eight hours to remove excess genipin. Before seeding with dhBMECs, channels were coated overnight with 100 μg mL^−1^ growth factor-reduced Matrigel in DMEM/F12. Derived hBMECs were seeded at 20 × 10^6^ cells mL^−1^ into collagen channels using hESCR1 media supplemented with 10 μM ROCK inhibitor Y27632 and allowed to adhere under static conditions for 30 minutes. ROCK inhibition was required to maintain cell adhesion, as previously reported^13^. After 24 hours, ROCK inhibitor was removed and microvessels were continually perfused with hECSR2. After cell seeding, microvessels were maintained under constant perfusion using a gravity driven flow system (inlet pressure of 5 cm H_2_O); the average endothelium shear stress was approximately 1 dyn cm^−2^ as calculated from Poiseuille’s Law.

### Quantifying microvessel structure and function

Permeability was measured using two different molecular weight solutes: 200 μM Lucifer yellow (CH dilithium salt; LY) and 2 μM Alexa Flour-647-conjugated 10 kDa dextran (Thermo Fisher). Phase contrast and fluorescence images were acquired every two minutes (NIS Elements) before (10 minutes; 5 frames) and after solute perfusion (60 minutes; 31 frames). At every time point, the protocol obtained six images: (1) a phase contrast image of the top of the microvessel (located and maintained by autofocus), (2–5) phase contrast and fluorescence images of the microvessel midplane, and (6) a phase contrast image of the bottom of the microvessel. Filter cubes (Chroma 39008 and Chroma 41008) were used to capture Lucifer yellow (20 ms exposure) and Alexa Fluor-647-conjugated dextran (200 ms exposure), respectively. Images were collected as ten adjacent frames using a 10× objective, resulting in a total image area of 8.18 mm × 0.67 mm.

ImageJ (NIH) was used to measure fluorescence intensity profiles over 36 frames for each of the ten adjacent frames. Permeability was calculated from (d/4)(1/ΔI)(dI/dt)_0_, where d is the diameter of the vessel, ΔI is the initial increase in total fluorescence intensity, and (dI/dt)_0_ is the rate of increase in total fluorescent intensity^11,69^. The rate of intensity increase (dI/dt) was measured over sixty minutes for Lucifer yellow and thirty minutes for 10 kDa dextran. Images were segmented into 10 adjacent regions-of-interest (ROIs), where the value of the ROI with minimum permeability is reported to exclude artifacts from interstitial dye entering the ECM from the inlet and outlet ports. The permeability detection limit was 1 × 10^−7^ cm s^−1^, as previously reported^11^.

The structure of BBB microvessels was monitored daily using phase contrast imaging. Survival of microvessels represents two criteria: (1) maintenance of perfusion, and (2) confluent endothelium attached to the collagen wall. Additionally, the ability of BBB microvessels to respond to fluid pressure was monitored using microscopy while the inlet and outlet height difference was changed. The inlet height was increased in increments of 5 cm from 5 cm to 15 cm and equilibrated for 5 minutes at each height. Phase contrast images were acquired at each pressure to record average microvessel diameter.

### Cryopreservation

Human iPSC-derived BMECs were cryopreserved and stored in liquid nitrogen on day eight as previously described^16^. dhBMECs were singularized in Accutase and then resuspended in 60% hESCR1, 30% fetal bovine serum (Sigma) and 10% dimethyl sulfoxide (Sigma). Cryovials were frozen in an isopropanol-filled freezing container (Mr. Frosty, Thermo Scientific) at -80 °C for 24 hours, then moved into liquid nitrogen for long-term storage. Cells were rapidly thawed in a water bath (~37 °C), centrifuged using fresh media, and then seeded onto transwells or glass, or seeded into microvessels as previously described. 10 μM ROCK inhibitor Y27632 (RI) was supplemented for the first 24 hours after thawing.

### Mechanical characterization of hydrogels and mouse brain

Hydrogels: Collagen hydrogels were fabricated by casting collagen I solutions between two glass plates separated by 2 millimeters. Collagen I solutions were gelled for 30 minutes at 37 °C. Some hydrogels were crosslinked with genipin and/or equilibrated with PBS as previously summarized. Hydrogel samples 10 mm in diameter and 2 mm in thickness were punched out and compressed at a rate of 0.25 mm s^−1^ using a tensile/compression tester (MTS Criterion). From the obtained stress-strain curve, the Young’s Modulus (Pa) was calculated as the best-fitted slope of the initial linear region (~5–12% strain). Compression testing could not be accurately conducted on 3 mg mL^−1^ collagen I hydrogels as they were physically unstable when unconstrained and collapsed by ~40% (height). The hydrogels of other conditions were stable and their heights varied less than 10%.

Mouse brain: 9–12 weeks old male and female BALB/c mice were euthanized, and had their brains harvested within one hour of compression tests. A coronal section of the whole brain was taken by cutting at the anterior and posterior coronal plane proximal to the pituitary gland (~3 mm thickness). Once the sample was sectioned, it was immediately staged on the compression tester for measurement. The compression plate was lowered to achieve initial contact with the flat tissue surface with minimum compressive force applied. The initial thickness of the tissue was then measured by the distance between the top and bottom (stage) plates for strain calculation. The sample was allowed to stabilize for one minute before compression testing. The tissue sample was then compressed at a rate of 2 mm min^−1^ using a tensile/compression tester (MTS Criterion). The Young’s modulus was calculated from the obtained stress-strain curve (~5–10% strain).

### Statistical analysis

Statistical testing was performed using Prism ver. 8 (GraphPad). TEER, permeability and Young’s moduli measurements are reported as mean ± standard error of the mean (SEM). A student’s unpaired t-test (two-tailed with unequal variance) or an analysis of variance (ANOVA) test were used for comparison between two or more than two groups, respectively. Reported p-values were multiplicity adjusted using a Tukey test. Analysis of Covariance (ANCOVA) was used to compare linear regression slopes. Differences were considered statistically significant for p < 0.05, with the following thresholds: *p < 0.05, **p < 0.01, ***p < 0.001.

## Supplementary information

Supplementary Information

## Data Availability

The raw and processed data from this study are available from the corresponding author on reasonable request.
