# Supplementary material for: Tissue-engineered blood-brain barrier models via directed differentiation of human induced pluripotent stem cells
Source: Sci Rep. 2019 Sep 27;9:13957. doi: 10.1038/s41598-019-50193-1 (PMC6764995; doi:10.1038/s41598-019-50193-1)
Supplement: Supplementary file 1 — Supplementary Information [file 41598_2019_50193_MOESM1_ESM.pdf]

SUPPLEMENTARY INFORMATION

Tissue-engineered blood-brain barrier models via directed differentiation of human induced pluripotent stem cells

Gabrielle Grifno,[1,2,+] Alanna Farrell,[1,2,+] Raleigh M. Linville,[1,2,+] Diego Arevalo,[1,2] Joo Ho Kim,[1,3] Luo Gu,[1,3] Peter C. Searson[1,2,3*]

[1] Institute for Nanobiotechnology, Johns Hopkins University, Baltimore, MD

[2] Department of Biomedical Engineering, Johns Hopkins University, Baltimore, MD

[3] Department of Materials Science and Engineering, Johns Hopkins University, Baltimore, MD

[+] These authors contributed equally

Supplementary Figure S1. Optimization of the directed differentiation of BC1-GFP iPSCs.

Supplementary Figure S2. Variability of directed differentiation across two iPSC lines.

Supplementary Table S1. Antibodies used in this study.

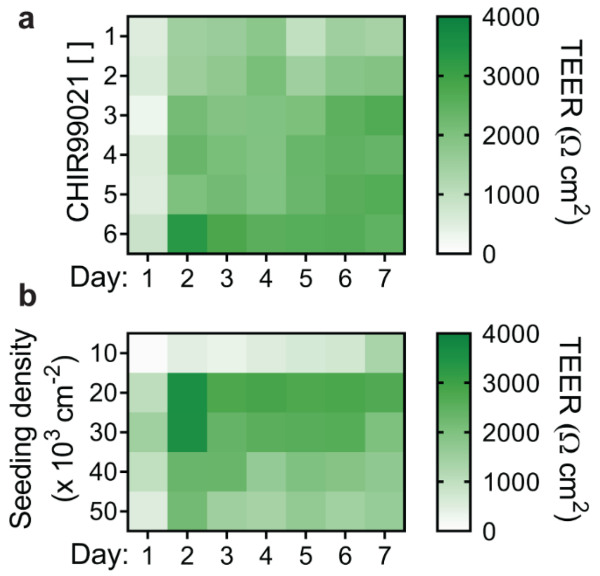

Supplementary Figure S1. Optimization of the directed differentiation of BC1-GFP iPSCs. Heatmap of transendothelial electrical resistance (TEER) values of dhBMECs as a function of CHIR concentration, seeding density, and time. (a) Time dependence of TEER for CHIR99021 concentrations of 1 – 6 μM (with an initial seeding density of 20,000 cells cm^{-2}). (b) Time dependence of TEER for initial seeding densities of 10 - 50 x 10^3 cells cm^{-2} (with a CHIR99021 concentration of 6 μM). Each square of the heatmaps represent the mean TEER across at least two independent differentiations.

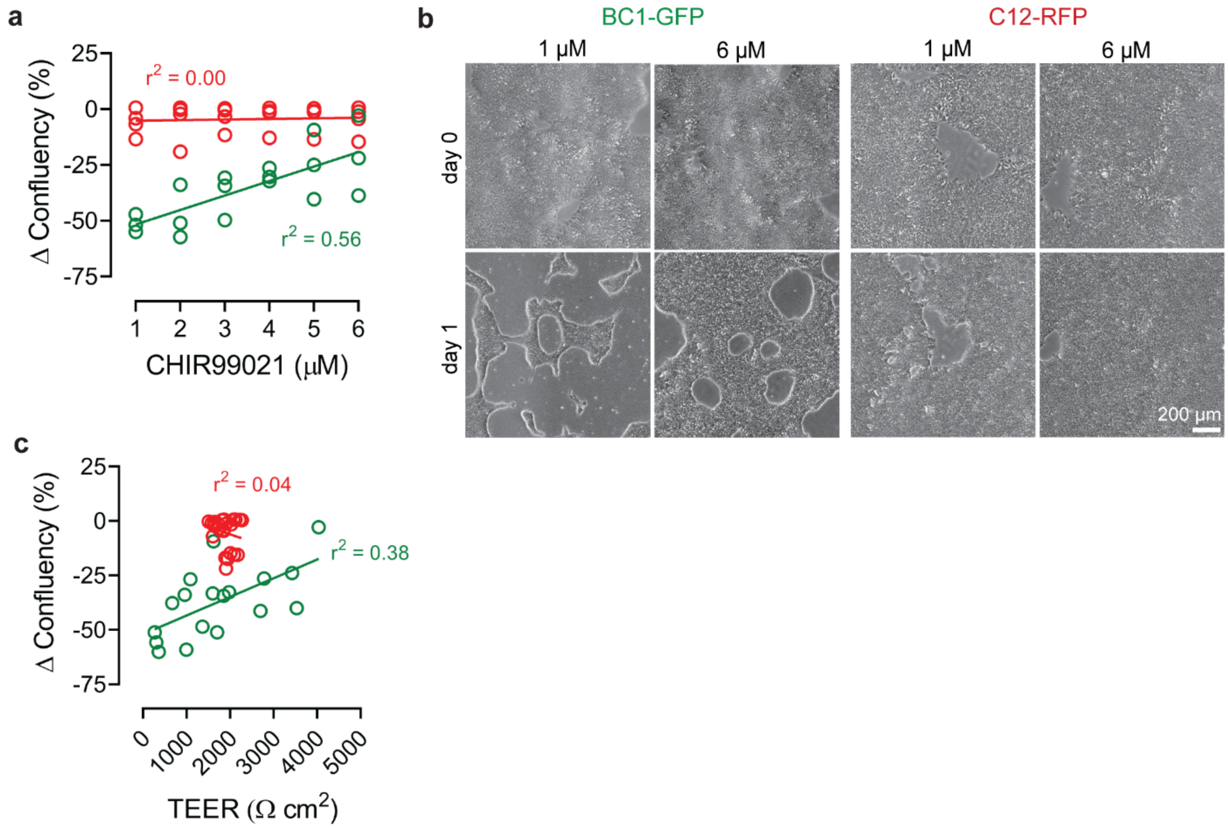

Supplementary Figure S2. Variability of directed differentiation across two iPSC lines. BC1-GFP and C12-RFPs were plated at 20,000 cells cm^{-2} and grown for three days. (a) Concentration of CHIR99021 versus change in iPSC confluency over 24-hour treatment period. Only BC1-GFPs display dose-dependent confluency changes. (b) Representative brightfield images of both iPSC lines treated with 1 and 6 μM CHIR99021 on day 0 (before treatment) and day 1 (24 hours after treatment). (c) TEER versus change in iPSC confluency over 24-hour treatment period for both iPSC lines. Lower changes in confluency are associated with higher TEER only for BC1-GFPs.

Supplementary Table S1. Antibodies used in this study.

	**Vendor**	**Species**	**Cat. No**	**Dilution**
CD31	ThermoFisher	Rabbit	402200	1:25
ZO-1	Invitrogen	Rabbit	402200	1:200
Claudin-5	Invitrogen	Mouse	35-2500	1:200
Occludin	Invitrogen	Rabbit	40-4700	1:100
GLUT1	Abcam	Rabbit	115730	1:200
P-gp	Sigma	Mouse	P7965	1:100
VE-cadherin	Santa Cruz	Mouse	Sc-9989	1:100
